# Age-Related Changes in Standing Sprint Start Performance and Posture in Sprinting

**DOI:** 10.3390/sports14080337

**Published:** 2026-08-05

**Authors:** Yoshiki Fujisawa, Ryohei Hayashi

**Affiliations:** 1 The Joint Graduate School in Science of School Education, Hyogo University of Teacher Education, Kato 673-1494, Japan; yoshiki.f.1206@icloud.com; 2 Faculty of Education, Gifu University, Gifu 501-1193, Japan

**Keywords:** sprint running, standing sprint start, acceleration, arm–leg coordination, physical education, motor development

## Abstract

The relationship between upper- and lower-limb positioning during standing sprint starts and acceleration performance across developmental stages remains unclear. The purpose of this study was to categorize the standing sprint start postures of participants ranging from elementary school to university students and to examine how these postures influence the time from the start signal to the first foot contact and the 5-m split time during a 50-m sprint. Starting postures were classified as Type A (same-side arm–leg alignment) or Type B (opposite-side arm–leg alignment). Two-way ANOVA revealed a significant main effect for posture type, indicating that Type B generally resulted in a shorter initial acceleration time than did Type A. Furthermore, a significant interaction effect was found for the 5-m split time for boys, with a particularly pronounced trend of decreasing times from lower elementary school to junior high school grades. These findings suggest that contralateral arm–leg positioning during standing sprint starts is associated with early acceleration performance and may reflect developmental changes in coordination relevant to sprint performance and coaching practice.

## 1. Introduction

Sprint running is the fastest form of human ground locomotion and is central to many sport and physical education contexts [1]. Sprint performance, which includes rapid acceleration and the ability to reach and maintain high maximum sprint speed, is an important determinant of performance in sprint-based sports across age groups [2,3]. In children, sprinting is also common during active play and physical education and represents a key locomotor component of fundamental movement skills [4,5].

Sprint performance is associated with competitive performance in various sports, such as soccer and rugby [2,6], and may influence team selection in competitive settings [6,7]. Because sprints often occur in such sports over short distances and brief time frames, early acceleration is particularly relevant to applied sport performance [8]. In track sprinting, performance is also strongly influenced by the start and the first steps of acceleration [9,10,11]. Thus, the ability to accelerate rapidly over short distances has broad relevance across sport, exercise, and physical education settings.

Studies of standing sprint starts have shown that compared to parallel starts, staggered false-step and split starts can improve initial sprint performance [12,13,14]. These advantages may reflect greater propulsive force, reduced braking, and a forward-leaning posture that places the body’s center of gravity ahead of the base of support [15]. In team sports, where athletes often adopt parallel stances to accommodate multidirectional movement, a backward false step may also support rapid acceleration [16].

Several studies have evaluated variables on short-distance acceleration during standing starts. For example, parallel starts result in longer 5-m and 10-m sprint times than split or false-step starts [17]. Similarly, starts involving a backward step have been associated with shorter 2.5-m and 5-m times than parallel starts [12,18]. Studies involving team-sport athletes have also reported faster short-distance sprint times and more favorable mechanical outputs after false-step or staggered false-step starts than after parallel or forward-step starts [13,15,19]. However, other findings suggest that a forward step may produce higher center-of-mass velocity during the first two steps and greater forward displacement than a false step [20]. Together, these findings indicate that acceleration performance during standing sprint starts may vary according to starting posture and step strategy.

In children, standing sprint start postures also vary across movement patterns. Among elementary school children, approximately half of males and females reportedly adopt a posture in which the forward arm and opposite leg are positioned forward during the standing sprint start [21]. Hioki et al. [21] reported that when children were briefly instructed to assume this contralateral posture at the start, their body’s center-of-gravity acceleration immediately improved. Because this performance enhancement occurred without comprehensive sprint training, it likely reflects a direct biomechanical advantage of the posture itself rather than other training adaptations. These results suggest that the contralateral posture might represent the optimal standing sprint start posture. However, because the study by Hioki and colleagues [21] included a small sample size and a limited age range, their findings may not fully capture developmental variation in standing sprint start posture from childhood through adolescence. Therefore, developmental trends in standing sprint start posture across a wide age range remain unclear.

Upper- and lower-limb coordination may also influence acceleration during the sprint start and early acceleration phases [22]. During the start phase, arm swing contributes to forward displacement of the body’s center of gravity and propulsive force generation. These findings suggest that arm–leg coordination during a standing sprint start may influence early acceleration. However, the role of arm positioning during standing sprint starts remains insufficiently characterized [22]. This limits the development of evidence-informed coaching guidance for standing sprint starts during early acceleration. Because sprint coaching often emphasizes consistent movement patterns, information on arm–leg positioning during standing sprint starts may have practical value [23]. Accordingly, clarifying upper- and lower-limb positioning during standing sprint starts may support both performance analysis and coaching practice.

Previous studies have examined the relationship between upper-limb movement during sprinting and sprint performance in participants from elementary school to high school. Hiruma and Kariyama [24] reported sex- and developmental-stage-specific differences in the relationship between arm-swing direction and sprint time, suggesting that upper-body movement is relevant to sprint performance. Their findings also indicate that upper-body positioning during the split start, a type of standing sprint start, may change with development. However, the relationship between upper-limb positioning during standing sprint starts and sprint performance remains unclear.

The purpose of this study was to categorize the standing sprint start postures of participants ranging from elementary school to university students and examine how these postures influenced the time from the start signal to the first foot contact (T1step) and the 5-m split time (T5m) during a 50-m sprint. Because contralateral coordination of the upper and lower limbs may support efficient force generation during early acceleration, a stance in which the forward arm is paired with the opposite forward leg may be advantageous for acceleration performance regardless of age [21]. We hypothesized that this relationship would be consistent across all developmental stages; specifically, Type B, in which the forward arm and opposite leg were positioned forward, would be associated with shorter T1step and T5m across all age groups than Type A, in which the forward arm and same-side leg were positioned forward.

## 2. Materials and Methods

### 2.1. Sample Selection

To estimate the sample size required for the primary outcome, i.e., 0–5-m sprint time, we conducted an a priori power analysis using G*Power for Mac (version 3.1.9.7; Institute for Experimental Psychology, Düsseldorf, Germany). The analysis was based on an independent-samples *t*-test with the following parameters: two-tailed test, effect size *d* = 0.8, α = 0.05, and power (1 − β) = 0.80. An effect size of *d* = 0.8 was selected as it represents a large effect according to Cohen’s conventional criteria [25] and was considered appropriate for detecting practically meaningful differences in sprint performance between the groups. This assumption is further supported by previous investigations comparing different standing sprint starts techniques, having consistently reported large effect sizes (*d* = 0.8) for initial sprint times and kinematic variables over short distances [20,26]. For instance, LeDune et al. [20] demonstrated very large effect sizes (Cohen’s *d* = 2.09–3.46) in kinematic differences between forward and false-step starts. Similarly, Duthie et al. [26] reported large to very large effects (effect size = 1.6–1.9) in 10-m sprint times between different starting techniques. Based on these assumptions, the required total number of participants was estimated to be 42 (i.e., 21 participants per group). While these large effect sizes were derived from experimental studies with manipulated starting techniques, smaller effect sizes (e.g., *d* = 0.2–0.5) are also considered practically meaningful in the context of sprint performance and may be more representative of differences observed in naturally selected postures. Because our actually recruited sample size in several age strata vastly exceeded the initial estimate of 42 participants, the study provided sufficient statistical power to detect these smaller, practically meaningful differences. However, the final sample size was determined by the number of eligible students who provided consent within the collaborating educational institutions. Because recruitment was constrained by the school setting and the availability of eligible participants, some subgroup analyses included fewer participants than the estimated target sample size of 21 participants per group. Specifically, these underpowered subgroups included Type A in male university students; Type B in male upper and lower elementary students; Types A and B in female university students; and Type B in female upper, middle, and lower elementary students. This limited statistical power must be considered when interpreting the non-significant findings in these specific strata. In particular, the lack of significant differences in sprint times between posture types among university participants may be attributable to an increased risk of Type II errors rather than a true absence of an effect. Thus, these non-significant results should be interpreted with caution, as noted in the Limitations section.

### 2.2. Participants

This study included 998 participants from Japan: 60 males and 60 females in the lower grades of elementary school, 56 males and 66 females in the middle grades of elementary school, 55 males and 57 females in the upper grades of elementary school, 113 males and 109 females in junior high school, 213 males and 137 females in high school, and 44 male and 28 female university students. Table 1 shows the participants’ physical characteristics, stratified by standing sprint start posture type (Type A and Type B) within each age group. Physical characteristics, including height and weight, were generally equivalent for both posture types within each specific age and sex subgroup, minimizing the potential confounding effect of physical maturation on sprint performance comparisons. For elementary, junior high, and high school students, the principal investigator explained the study outline during physical education classes at each institution and distributed information leaflets and consent forms to students and their parents or guardians. Elementary school students were grouped according to the framework adopted by Tanaka et al. [27], which reflects standard Japanese physical education categories: lower grades (1st and 2nd grades), middle grades (3rd and 4th grades), and upper grades (5th and 6th grades). The inclusion criteria were no prior specialized sprint training, regular participation in physical education classes, ability to complete a 50-m sprint, and absence of medical conditions that would preclude participation in the experimental trials. Exclusion criteria were the use of medications that could affect athletic performance and the presence of orthopedic limitations.

### 2.3. Experimental Procedures

The experiment was conducted during teacher-led physical education and practical sports classes. To reduce the risk of sprint-related muscle, tendon, or joint injuries, all participants performed a standardized warm-up consisting of 3 min of running and dynamic stretching during their regular physical education classes. Then, they performed three 50-m sprints at submaximal intensity [19] to familiarize themselves with the standing start procedure and stabilize their movement patterns. After the warm-up, participants performed one 50-m sprint at maximum effort. A single maximum-effort trial was selected to accommodate the strict time constraints of regular physical education classes and to prioritize the ecological validity of evaluating a large sample (*n* = 998) within a real-world educational setting. Because the study focused on group-level developmental trends rather than individual athletic profiling, the substantial sample size mitigated the influence of individual trial-to-trial variability. Because sprint times were calculated using video analysis, participants received no feedback on sprint performance. Participants were instructed to avoid vigorous exercise for 24 h before the experiment [15].

### 2.4. Sprint Performance Measurement

To evaluate sprint performance, participants wore running shoes and performed a maximum-effort 50-m sprint from a standing start with both feet on the ground on the straight sections of outdoor dirt tracks at the participating educational institutions. Participants were verbally instructed to run through the finish line to avoid deceleration before completing the sprint. After participants were positioned at the starting line, they were instructed to stop completely and assume a ready position on the “Set” command; the trial then began with a starting signal from an electronic whistle (RA0010-B; Molten, Hiroshima, Japan). Participants were informed in advance that the starting signal would be an electronic tone, and trials were conducted only after confirming that participants could start normally. Sprint trials were conducted under nearly windless conditions and were not performed on rainy days or when the temperature was below 10 °C. For the standing sprint start, participants adopted a stance in which only their feet touched the ground [14], with no other restrictions imposed. If a participant was not completely stationary at the “Set” command or made a false start, the start was repeated. Attempts were considered unsuccessful if the participant fell or slipped during the sprint. Participants were instructed to perform at maximum effort. If a trial was deemed unsuccessful, a 5-min rest period was provided before the participant repeated the trial.

### 2.5. Evaluation of the Standing Sprint Start

The types of standing sprint start postures were classified using an observational assessment method based on Hiruma and Kariyama [24]. This method has been used to analyze arm swing in elite sprinters [28] and standing sprint start posture in children [21]. Using the recorded footage, five assessors classified starting postures according to arm and leg positions at the “Set” moment immediately before the standing sprint start.

The assessors comprised one university faculty member specializing in track and field, with national-level coaching experience and a PhD in coaching science; three graduate students specializing in track and field; and one undergraduate student specializing in track and field. Participants were classified into two posture types: Type A and Type B. The assessments of the five assessors were then compiled, and classifications agreed on by at least three assessors were adopted as the final classifications. If two or fewer assessors agreed on the initial classification, the video was replayed and reviewed until at least three assessors reached consensus.

### 2.6. Measures

All experimental trials were recorded using one high-speed digital video camera (GC-LJ20B; SPORTS SENSING, Fukuoka, Japan). The camera was used to observe arm and leg positions during the standing sprint starts and to measure T1step [29] and T5m [12,15,18,19]. A marker was placed 1 m behind the starting line, and the 3-m mark was defined from this point. The camera was positioned 45.5 m perpendicular to the track at the extension of this 3-m mark and set to continuous recording at 300 Hz. The 5-m time was used because differences in sprint performance associated with starting method are evident at 5 m but appear to diminish by 25 m and 50 m [15,19]. T1step and T5m during the 50-m sprint were measured and analyzed using a video digitizing system (Frame-DIAS; DKH, Tokyo, Japan). The reference frame was defined as the frame in which the omnidirectional light source (PH-145; DKH), installed near the starter and synchronized with the starting signal, was illuminated. The frames corresponding to the first foot contact and the passage of the torso over the 5-m reference mark were then identified. T1step and T5m were calculated from the time differences between these frames.

### 2.7. Statistical Analysis

All statistical analyses were performed using IBM SPSS Statistics version 25.0 (IBM, Armonk, NY, USA). Normality was assessed using the Kolmogorov–Smirnov test for T1step and T5m in each combination of sex (males and females), age group, and posture type (Type A and Type B). The results of the Kolmogorov–Smirnov test indicated that more than 90% of the data followed a normal distribution; therefore, parametric tests were adopted to ensure consistency in the analytical approach [30]. In addition to the Kolmogorov–Smirnov test, visual inspections of histograms and Q–Q plots were performed to confirm the normality of the data, especially for subgroups with smaller sample sizes (e.g., female university participants). To assess the inter-rater reliability of the posture classification, Fleiss’ kappa coefficient (*κ*) was calculated from the independent classifications made by the five raters [31]. The interpretation of *κ* values followed the criteria proposed by Landis and Koch [32]: poor (<0.00), slight agreement (0.00–0.20), fair agreement (0.21–0.40), moderate agreement (0.41–0.60), substantial agreement (0.61–0.80), and almost perfect agreement (0.81–1.00). Contingency tables were constructed for age group and standing sprint start posture type separately for males and females. Pearson’s chi-square (χ^2^) test was used to examine associations between age group and posture type, and adjusted standardized residual analyses were performed to identify cells contributing significantly to the observed associations. A two-way analysis of variance (ANOVA; age group × posture type) was conducted to examine the effects of developmental stage and standing sprint start posture on T1step and T5m. Post hoc pairwise comparisons with Bonferroni correction were used to assess differences between posture types within each age group. To examine the strength of the relationship between T1step and T5m, Pearson’s product–moment correlation coefficient (*r*) was used. Cohen’s *d* was calculated to quantify the magnitude of between-group differences. Effect sizes were interpreted as trivial (≤0.2), small (>0.2 to 0.5), medium (>0.5 to 0.8), large (>0.8 to 1.3), and very large (>1.3) [25]. The significance level was set at *p* < 0.05. Data are presented as the mean ± standard deviation.

## 3. Results

The initial independent classifications by the five assessors showed almost perfect agreement, with a *κ* of 0.90 (*p* < 0.01). This indicates highly reliable posture classification before the majority-rule consensus was applied.

Table 2 presents the cross-tabulation of age group and standing sprint start posture type in male participants. A chi-square test revealed a significant association between age group and posture type (χ^2^ = 17.95, *p* < 0.01). Subsequent adjusted standardized residual analyses indicated that the observed frequency of Type B was significantly higher than expected among university students, whereas the observed frequency of Type A was significantly higher than expected among males in the upper and lower elementary school groups. Conversely, the observed frequency of Type A was significantly lower than expected among university students, and the observed frequency of Type B was significantly lower than expected among males in the upper and lower elementary school groups.

Table 3 presents the cross-tabulation of age group and standing sprint start posture type in female participants. A chi-square test revealed a significant association between age group and posture type (χ^2^ = 34.13, *p* < 0.01). Subsequent adjusted standardized residual analyses indicated that the observed frequency of Type B was significantly higher than expected among female university and high school students, whereas the observed frequency of Type A was significantly higher than expected among females in the middle and lower elementary school groups. Conversely, the observed frequency of Type A was significantly lower than expected among female university and high school students, and the observed frequency of Type B was significantly lower than expected among females in the middle and lower elementary school groups.

Table 4 compares T1step and T5m between posture types among male participants in each age group. A two-way ANOVA for T1step revealed no significant interaction (*p* = 0.096) but showed significant main effects of age group (*p* < 0.01) and posture type (*p* < 0.01). For T5m, a significant interaction was found (*p* = 0.031), as well as significant main effects of age group (*p* < 0.01) and posture type (*p* < 0.01). Post hoc analyses for T5m revealed that Type B was associated with significantly shorter times than Type A among males in the lower, middle, and upper elementary grades, as well as those in junior high school (*p* < 0.05 and *p* < 0.01), with moderate effect sizes (Cohen’s *d* = 0.54–0.77).

Table 5 compares T1step and T5m between posture types among female participants in each age group. A two-way ANOVA for T1step revealed no significant interaction (*p* = 0.060) but showed significant main effects of age group (*p* < 0.01) and posture type (*p* < 0.01). Similarly, for T5m, no significant interaction was detected (*p* = 0.409), but significant main effects of age group (*p* < 0.01) and posture type (*p* < 0.01) were observed.

Furthermore, correlation analyses revealed significant positive correlations between T1step and T5m across all age and sex subgroups for both posture types. Significant positive correlations were observed in male participants for both Type A (*r* = 0.752–0.899, *p* < 0.01) and Type B (*r* = 0.611–0.911, *p* < 0.01), as well as in female participants for both Type A (*r* = 0.310–0.951, *p* < 0.05) and Type B (*r* = 0.797–0.921, *p* < 0.01).

## 4. Discussion

The purpose of this study was to categorize the standing sprint start postures of participants ranging from elementary school to university students and examine how these postures influenced T1step and T5m during a 50-m sprint. The results of two-way ANOVA revealed significant main effects of posture type on T1step for both sexes and T5m for female students, indicating that Type B generally yielded faster early acceleration times than did Type A across developmental stages. Furthermore, a significant interaction was observed between age group and posture type for T5m among male students, underscoring a nuanced, age-dependent divergence in performance. These findings are consistent with the hypothesis that Type B is associated with better early acceleration performance than Type A during childhood and adolescence. The results suggest that relative arm–leg positioning during a standing sprint start may be relevant to early acceleration performance.

These findings are consistent with the results of Hioki et al. [21], who reported greater acceleration in the Type B posture than in the Type A posture. A key finding of the present study was that Type B demonstrated higher acceleration performance than did Type A across developmental stages. This superiority was particularly pronounced in the early acceleration phase of T5m among male students from lower elementary grades through junior high school.

**Figure 1:**
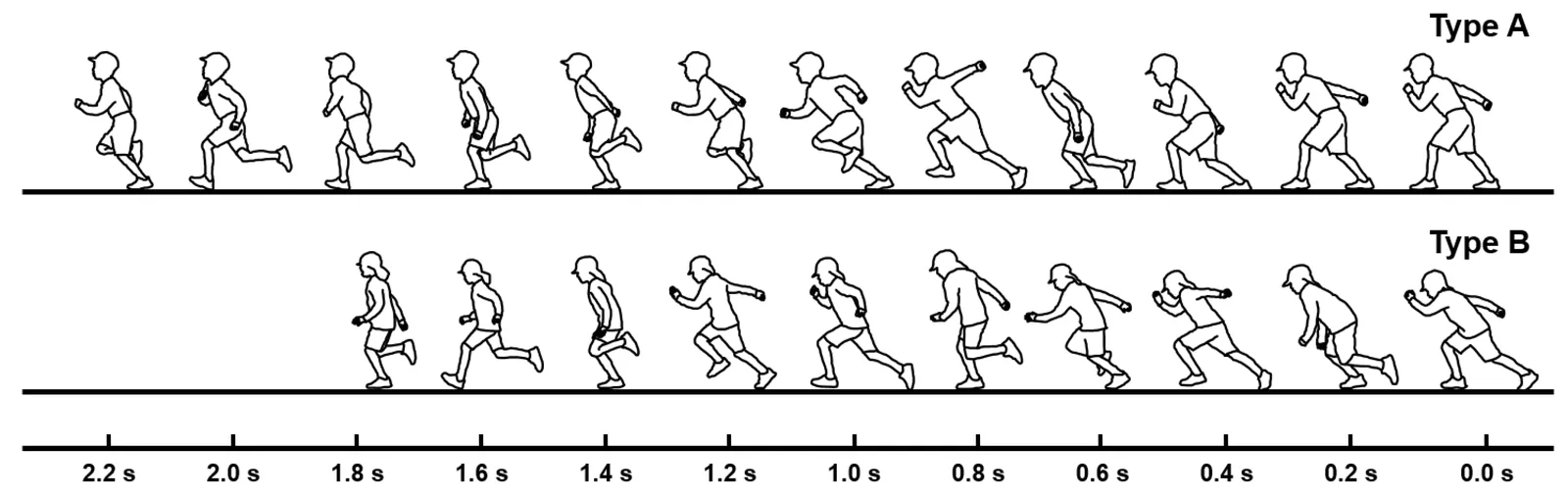
Representative starting postures and sprint motion for Types A and B up to the 5-m mark.

In sprinting, the arms coordinate with the opposite leg and may contribute to horizontal propulsion [33]. Arm swing may help maintain balance by offsetting angular momentum generated by pelvic rotation and may enhance forward propulsion by increasing stride length and ground reaction force [34]. While the observational nature of this study precludes causal inferences, the observed differences in T1step and T5m might be explained by differences in movement efficiency. Specifically, Type B might be associated with a smoother transition into the contralateral arm–leg coordination characteristic of sprinting immediately after the starting signal. In contrast, participants using Type A might need an additional arm movement after the starting signal to achieve the contralateral coordination pattern observed in Type B, which may delay horizontal displacement at the start and contribute to longer T1step and T5m (Figure 1). Alternatively, because the starting posture was selected based on the participant’s preference, it is equally possible that participants who already possessed superior coordination or sprint ability naturally gravitated toward adopting the Type B posture, rather than the posture itself causing the performance advantage. However, it must be emphasized that these movement differences are speculative. Because the starting posture was not experimentally assigned, we cannot determine whether the Type B posture directly causes shorter sprint times or whether individuals with inherently better motor coordination simply prefer this posture. The movement differences described above may partly explain the observed differences in T1step and T5m.

Frost et al. [12] compared sprint times of up to 5 m across parallel, false-step, and split starts. These authors found that false-step and split starts produced faster 0–2.5-m times than parallel starts when reaction time was excluded. Maintaining an early advantage in the 0–2.5-m segment may improve the overall 0–5-m time [12,15]. To explore this relationship within our dataset, we examined the correlations between T1step and T5m. We found significant positive correlations across all age and sex subgroups for both posture types. Although the cross-sectional design of our study cannot establish a direct causal sequence, these significant correlations support the biomechanical plausibility that the shorter T1step observed in Type B is closely associated with the shorter T5m in several age groups. Therefore, the shorter T1step observed in Type B may have contributed to the shorter T5m in several age groups. However, it is important to note that T1step in our study was measured from the auditory starting signal to the first foot contact. As such, this measurement inherently encompasses both the auditory reaction time and the actual movement execution time. Consequently, we cannot definitively determine whether the shorter T1step associated with Type B is driven solely by the biomechanical efficiency of the posture itself or if it partially reflects faster auditory reaction times among participants who selected Type B. Separating cognitive reaction time from biomechanical movement time was not possible with our current video-based assessment, leaving this specific mechanism as a subject for future laboratory-based investigations.

Among elementary and junior high school students, Type A was selected more frequently than Type B (Table 2 and Table 3). In contrast, university students more frequently selected Type B (Table 2 and Table 3). Because neuromuscular coordination and motor skills differ across developmental stages [35], the contralateral arm–leg coordination patterns used in sprinting might be less common in younger populations. This developmental factor may partly explain why younger participants more often selected Type A, in which the forward arm and same-side leg were positioned forward. The higher prevalence of Type B among older participants suggests that this posture is more characteristic of later developmental stages. These cross-sectional differences between age groups might reflect variations in physical maturation, cumulative sprinting experience, and coaching exposure across the different cohorts, rather than intra-individual developmental changes. However, because sprinting experience and coaching exposure were not directly measured in the present study, these explanations remain speculative. Future investigations are warranted to determine the specific effects of these experiential factors on standing sprint start coordination.

**Figure 2:**
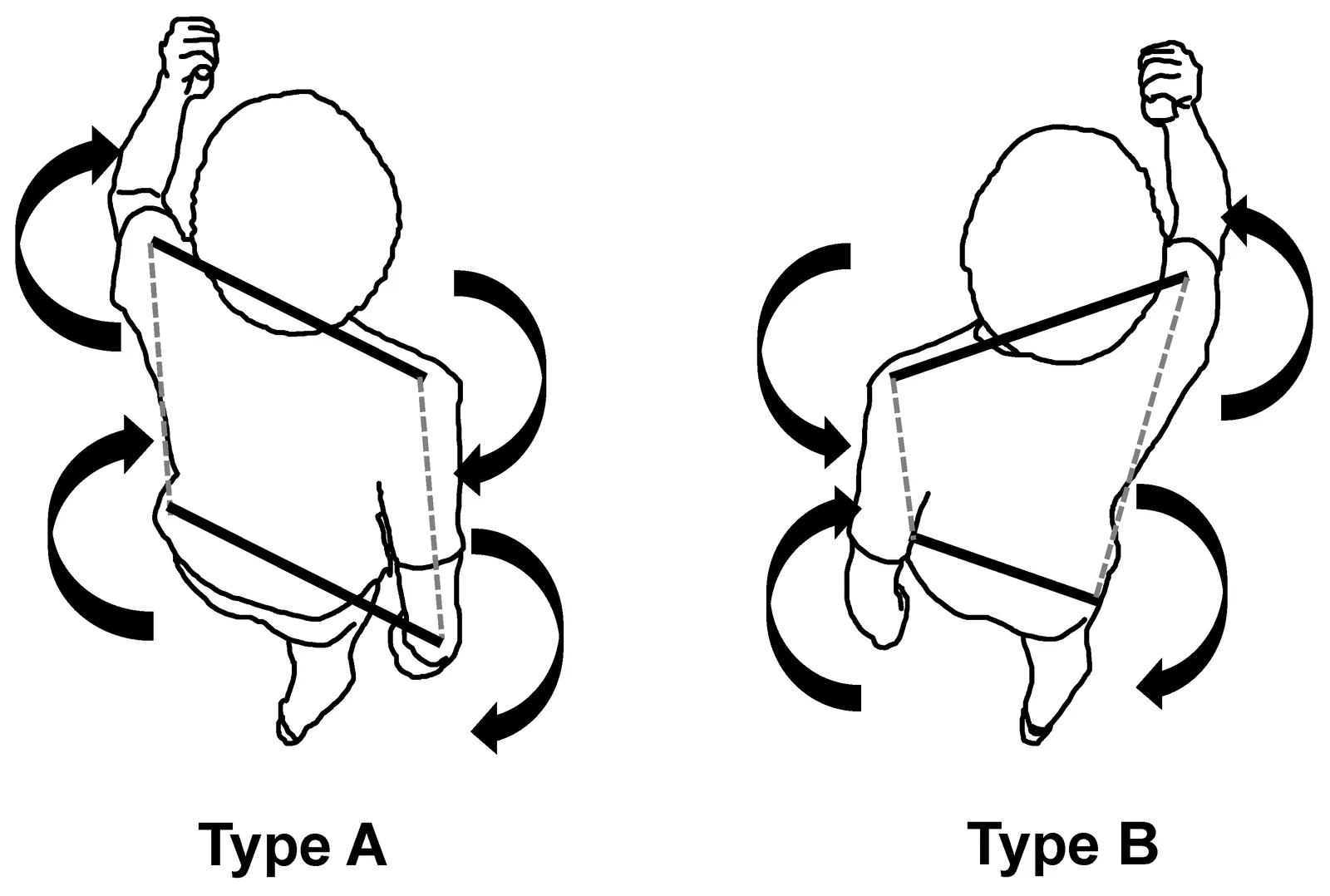
Relationship between the acromial line and pelvic orientation in Types A (**left**) and B (**right**). A line connects the shoulders and pelvis in the transverse plane, with curved arrows indicating their direction of rotation.

Type A may reflect a posture in which trunk and pelvic rotation are more closely aligned, with relatively limited axial twisting. In contrast, Type B may involve greater dissociation between trunk and pelvic orientation. Studies of children’s locomotion have reported that young children may use an “en bloc” coordination strategy, moving the head, trunk, and pelvis as a single unit rather than controlling these segments independently [36]. This strategy may simplify postural and balance control by reducing the number of joint degrees of freedom that must be controlled simultaneously [36]. Therefore, children may unconsciously prioritize postural stability and the avoidance of falling over the biomechanical pursuit of speed, leading to the natural selection of Type A. Walking studies also suggest that upper- and lower-limb coordination and trunk–pelvis control strategies change across developmental stages [35]. For example, Mani et al. [37] reported that coordination between limb swing and trunk movement changes from early to later childhood. By approximately 9–10 years of age, children may show more adult-like coordination patterns in which limb swing and trunk movement are controlled more flexibly according to task demands. However, our data demonstrated that even in junior high school, a high proportion of students continued to select Type A. This indicates a “developmental lag”; while motor control for daily routine movements like walking matures relatively early, it does not immediately translate into the natural acquisition of optimal coordination patterns (Type B) for high-load, non-routine tasks like maximum-effort sprint starts. Thus, Type A may require less independent segmental control than Type B, which may help explain its greater prevalence among younger participants (Figure 2). Coordinated trunk and pelvic orientation before the first step might provide a favorable preparatory state for forward propulsion and postural stability. Nevertheless, this clear discrepancy between the posture naturally selected by children (Type A) and the posture that actually yields faster sprint times (Type B) provides a strong rationale for intentional pedagogical intervention. Rather than waiting for students to naturally overcome this developmental lag and independently acquire complex coordination, educators can provide simple, static instructions to adopt the Type B posture. This direct coaching approach can serve as a highly effective streamlined approach to guide children toward greater biomechanical efficiency. However, it is important to emphasize that because these specific kinematic variables were not measured in the current study, the underlying mechanisms remain theoretical explanations based on developmental motor control literature. Future biomechanical investigations are required to directly confirm the presence and specific effects of trunk–pelvis dissociation and “en bloc” strategies during standing sprint starts.

Overall, the significant main effects of posture type demonstrated that Type B influenced shorter sprint times, suggesting that contralateral arm–leg positioning during standing sprint starts is generally advantageous for early acceleration performance across developmental stages. Additionally, the significant interaction observed for T5m among male students indicates that this pattern is particularly impactful from lower elementary grades through junior high school, suggesting that coordination patterns for standing sprint starts may emerge relatively early in male individuals. These findings may inform physical education and coaching by highlighting contralateral arm–leg positioning as a potentially useful focus during standing sprint start instruction.

This study has several limitations. First, due to the cross-sectional and observational design, causality cannot be established, and age-related differences cannot be interpreted as intra-individual longitudinal changes. Children with better baseline sprint ability might naturally gravitate toward Type B. Future experimental and longitudinal studies are needed. Second, our video-based assessments lacked detailed kinematic and kinetic analyses. Thus, the exact biomechanical mechanisms underlying the performance differences remain speculative and require future laboratory-based investigations. Third, while the overall sample size was adequate, stratifying the data into 12 age-by-sex subgroups resulted in small per-cell sample sizes in certain strata (e.g., female and university groups), increasing the risk of Type II errors in these specific comparisons. Fourth, because the participants were general students, the findings may not directly generalize to track-and-field athletes or multidirectional team-sport athletes. Finally, although the large group-level sample size mitigated overall trial-to-trial variability, reliance on a single maximum-effort trial inherently introduces individual-level measurement noise, which could potentially attenuate the observed differences between posture types.

Despite these limitations, this study provides age- and sex-specific data on the association between standing sprint start posture and early acceleration performance in participants from elementary school to university. Importantly, these results were obtained using a practical and broadly applicable observational assessment method, which can be readily implemented by physical education teachers and coaches involved in the sprint development of children. This approach might facilitate the identification of standing sprint start characteristics and support evidence-informed instruction in educational and youth sport settings.

## 5. Conclusions

The present study demonstrated significant main effects of posture type, indicating that Type B is generally associated with shorter early acceleration times (T1step and T5m) than is Type A across developmental stages. Furthermore, the significant interaction for T5m among male students indicates that this performance advantage is particularly pronounced from lower elementary grades through junior high school. These observational findings suggest that contralateral arm–leg positioning during standing sprint starts is broadly relevant to early acceleration performance, and they may guide effective physical education and sports coaching by helping instructors identify contralateral arm–leg positioning as a potentially useful focus during standing sprint start instruction. However, further longitudinal and interventional studies are needed to determine whether actively teaching contralateral arm–leg positioning improves standing sprint start performance across developmental stages. Such evidence should be established before recommending widespread changes to coaching and instructional practices.

## Figures and Tables

**Table 1:** Physical characteristics of participants in each age group (mean ± SD).

Males						
	University (*n* = 44)	High School (*n* = 213)	Junior High (*n* = 113)	Upper (*n* = 55)	Middle (*n* = 56)	Lower (*n* = 60)
Type A						
Age (y)	19.38 ± 0.72	16.74 ± 0.95	13.53 ± 0.99	9.08 ± 0.70	8.83 ± 0.75	7.12 ± 0.76
Height (m)	1.71 ± 0.03	1.69 ± 0.05	1.63 ± 0.08	1.50 ± 0.09	1.34 ± 0.06	1.25 ± 0.05
Weight (kg)	62.02 ± 5.77	61.28 ± 7.51	50.37 ± 8.14	39.94 ± 7.02	30.85 ± 5.47	24.50 ± 3.03
Type B						
Age (y)	19.36 ± 0.68	16.47 ± 0.97	13.45 ± 1.01	9.19 ± 0.75	9.08 ± 0.74	6.94 ± 0.75
Height (m)	1.70 ± 0.06	1.69 ± 0.06	1.57 ± 0.10	1.50 ± 0.10	1.35 ± 0.06	1.26 ± 0.04
Weight (kg)	61.82 ± 7.47	60.68 ± 11.50	48.09 ± 7.85	43.61 ± 4.33	33.80 ± 7.08	25.06 ± 2.56
Females						
	University (*n* = 28)	High school (*n* = 137)	Junior high (*n* = 109)	Upper (*n* = 57)	Middle (*n* = 66)	Lower (*n* = 60)
Type A						
Age (y)	19.38 ± 0.74	16.91 ± 0.95	13.42 ± 1.05	9.03 ± 0.69	8.88 ± 0.67	7.02 ± 0.76
Height (m)	1.57 ± 0.04	1.56 ± 0.08	1.53 ± 0.05	1.45 ± 0.08	1.34 ± 0.06	1.21 ± 0.02
Weight (kg)	52.75 ± 4.95	50.62 ± 8.22	47.94 ± 7.22	39.44 ± 7.63	29.54 ± 5.25	23.29 ± 3.47
Type B						
Age (y)	19.45 ± 0.69	16.59 ± 1.00	13.38 ± 0.88	9.10 ± 0.72	9.00 ± 0.71	7.17 ± 0.72
Height (m)	1.61 ± 0.05	1.58 ± 0.07	1.53 ± 0.06	1.46 ± 0.06	1.36 ± 0.07	1.24 ± 0.04
Weight (kg)	52.05 ± 4.97	50.96 ± 6.87	48.83 ± 7.25	39.07 ± 3.58	30.71 ± 7.48	25.58 ± 4.42

Junior high: junior high school, Lower: lower grade in elementary school, Middle: middle grade in elementary school, Upper: upper grade in elementary school, SD: standard deviation.

**Table 2:** Cross-tabulation of age and standing sprint start posture type in male participants.

Grade		Type of Standing Sprint Start Posture		Total
		Type A	Type B	
University	Observed frequency	16	28	44
	Adjusted residual	−2.9 **	2.9 **	
High school	Observed frequency	118	95	213
	Adjusted residual	−0.6	0.6	
Junior high	Observed frequency	62	51	113
	Adjusted residual	−0.5	0.5	
Upper	Observed frequency	39	16	55
	Adjusted residual	2.2 *	−2.2 *	
Middle	Observed frequency	30	26	56
	Adjusted residual	−0.5	0.5	
Lower	Observed frequency	43	17	60
	Adjusted residual	2.4 *	−2.4 *	
Total				541

Lower: lower grade in elementary school, Middle: middle grade in elementary school, Upper: upper grade in elementary school. * *p* < 0.05; ** *p* < 0.01.

**Table 3:** Cross-tabulation of age and standing sprint start posture type in female participants.

		Type of Standing Sprint Start Posture		Total
		Type A	Type B	
University	Observed frequency	8	20	28
	Adjusted residual	−3.4 **	3.4 **	
High school	Observed frequency	69	68	137
	Adjusted residual	−2.5 **	2.5 **	
Junior high	Observed frequency	59	50	109
	Adjusted residual	−1.2	1.2	
Upper	Observed frequency	37	20	57
	Adjusted residual	1.0	−1.0	
Middle	Observed frequency	49	17	66
	Adjusted residual	2.7 **	−2.7 **	
Lower	Observed frequency	48	12	60
	Adjusted residual	3.5 **	−3.5 **	
Total				457

Lower: lower grade in elementary school, Middle: middle grade in elementary school, Upper: upper grade in elementary school. ** *p* < 0.01.

**Table 4:** Sprint times by standing sprint start posture type among male participants in each age group (mean ± SD).

T1step							
	Type A (s)	95% CI	Type B (s)	95% CI	Difference		
					Type	Age	
University	0.70 ± 0.13	0.63–0.77	0.65 ± 0.11	0.60–0.69	A > B **	U < H * J, Up < H ** U, J, Up, M < L **	
High school	0.76 ± 0.12	0.74–0.79	0.73 ± 0.13	0.70–0.76			
Junior high	0.70 ± 0.10	0.68–0.73	0.63 ± 0.13	0.59–0.66			
Upper	0.72 ± 0.14	0.68–0.77	0.60 ± 0.14	0.53–0.68			
Middle	0.75 ± 0.16	0.69–0.81	0.64 ± 0.14	0.58–0.69			
Lower	0.84 ± 0.11	0.80–0.87	0.73 ± 0.15	0.65–0.81			
T5m							
	Type A (s)	95% CI	Type B (s)	95% CI	Difference		
					Type	Age (Type A)	Age (Type B)
University	1.56 ± 0.14	1.49–1.64	1.50 ± 0.15	1.44–1.56	−	U, H, J, Up, M < L ** U, H, J < M ** U, H, J < U **	U, H, J, Up, M < L ** H < M * U, J < M ** U < Up * U, J < H **
High school	1.67 ± 0.13	1.65–1.70	1.66 ± 0.14	1.63–1.69	−		
Junior high	1.64 ± 0.14	1.61–1.68	1.57 ± 0.15	1.53–1.61	A > B **		
Upper	1.78 ± 0.19	1.72–1.84	1.64 ± 0.14	1.57–1.72	A > B **		
Middle	1.87 ± 0.16	1.81–1.93	1.77 ± 0.17	1.70–1.84	A > B **		
Lower	2.07 ± 0.14	2.02–2.11	1.95 ± 0.18	1.86–2.04	A > B **		

Junior high (J): junior high school, Lower (L): lower grade in elementary school, Middle (M): middle grade in elementary school, Upper (Up): upper grade in elementary school, H: high school, U: university, T1step: start to first foot contact time, T5m: 0–5-m time, CI: confidence interval, SD: standard deviation. * *p* < 0.05; ** *p* < 0.01.

**Table 5:** Sprint times by standing sprint start posture type among female participants in each age group (mean ± SD).

T1step						
	Type A (s)	95% CI	Type B (s)	95% CI	Difference	
					Type	Age
University	0.73 ± 0.11	0.63–0.82	0.65 ± 0.12	0.60–0.71	A > B **	J < H ** J < M **
High school	0.78 ± 0.10	0.75–0.80	0.76 ± 0.09	0.73–0.78		
Junior high	0.74 ± 0.15	0.70–0.77	0.66 ± 0.16	0.62–0.71		
Upper	0.80 ± 0.13	0.76–0.85	0.69 ± 0.15	0.63–0.76		
Middle	0.81 ± 0.15	0.76–0.85	0.77 ± 0.12	0.71–0.83		
Lower	0.80 ± 0.12	0.76–0.83	0.64 ± 0.22	0.50–0.78		
T5m						
	Type A (s)	95% CI	Type B (s)	95% CI	Difference	
					Type	Age
University	1.73 ± 0.14	1.62–1.84	1.71 ± 0.18	1.62–1.79	A > B **	U, H, J, Up < L ** U < M * J < M **
High school	1.86 ± 0.17	1.82–1.90	1.82 ± 0.13	1.79–1.86		
Junior high	1.79 ± 0.16	1.75–1.83	1.72 ± 0.18	1.67–1.77		
Upper	1.87 ± 0.18	1.81–1.93	1.77 ± 0.12	1.71–1.82		
Middle	1.95 ± 0.17	1.90–2.00	1.92 ± 0.13	1.85–1.98		
Lower	2.14 ± 0.61	1.96–2.32	1.91 ± 0.22	1.77–2.06		

Junior high (J): junior high school, Lower (L): lower grade in elementary school, Middle (M): middle grade in elementary school, Upper (Up): upper grade in elementary school, H: high school, U: university, T1step: start to first foot contact time, T5m: 0–5-m time, SD: standard deviation. * *p* < 0.05; ** *p* < 0.01.

## Data Availability

The original contributions presented in this study are included in the article. Further inquiries can be directed to the corresponding author.

## Abbreviations

The following abbreviations are used in this manuscript:

ANOVA	Analysis of variance
T1step	Time from the start signal to first foot contact
T5m	Time to the 5-m mark
